# Comparison of the Chinese bamboo partridge and red Junglefowl genome sequences highlights the importance of demography in genome evolution

**DOI:** 10.1186/s12864-018-4711-0

**Published:** 2018-05-08

**Authors:** G. P. Tiley, R. T. Kimball, E. L. Braun, J. G. Burleigh

**Affiliations:** 1 Department of Biology, University of Florida, Gainesville, FL 32611 USA; 2 Department of Biology, Duke University, Durham, NC 27708 USA

**Keywords:** Galliforms, Phasianidae, Nonsynonymous to synonymous substitution rate ratio, Selection, Effective population size, Alignment uncertainty

## Abstract

**Background:**

Recent large-scale whole genome sequencing efforts in birds have elucidated broad patterns of avian phylogeny and genome evolution. However, despite the great interest in economically important phasianids like *Gallus gallus* (Red Junglefowl, the progenitor of the chicken), we know little about the genomes of closely related species. *Gallus gallus* is highly sexually dichromatic and polygynous, but its sister genus, *Bambusicola*, is smaller, sexually monomorphic, and monogamous with biparental care. We sequenced the genome of *Bambusicola thoracicus* (Chinese Bamboo Partridge) using a single insert library to test hypotheses about genome evolution in galliforms. Selection acting at the phenotypic level could result in more evidence of positive selection in the *Gallus* genome than in *Bambusicola*. However, the historical range size of *Bambusicola* was likely smaller than *Gallus*, and demographic effects could lead to higher rates of nonsynonymous substitution in *Bambusicola* than in *Gallus*.

**Results:**

We generated a genome assembly suitable for evolutionary analyses. We examined the impact of selection on coding regions by examining shifts in the average nonsynonymous to synonymous rate ratio (*dN/dS*) and the proportion of sites subject to episodic positive selection. We observed elevated *dN/dS* in *Bambusicola* relative to *Gallus,* which is consistent with our hypothesis that demographic effects may be important drivers of genome evolution in *Bambusicola*. We also demonstrated that alignment error can greatly inflate estimates of the number of genes that experienced episodic positive selection and heterogeneity in *dN/dS*. However, overall patterns of molecular evolution were robust to alignment uncertainty. *Bambusicola thoracicus* has higher estimates of heterozygosity than *Gallus gallus*, possibly due to migration events over the past 100,000 years.

**Conclusions:**

Our results emphasized the importance of demographic processes in generating the patterns of variation between *Bambusicola* and *Gallus*. We also demonstrated that genome assemblies generated using a single library can provide valuable insights into avian evolutionary history and found that it is important to account for alignment uncertainty in evolutionary inferences from draft genomes.

**Electronic supplementary material:**

The online version of this article (10.1186/s12864-018-4711-0) contains supplementary material, which is available to authorized users.

## Background

The availability of whole-genome sequences from phylogenetically diverse bird species has provided broad insights into avian evolution [1, 2]. Galliformes, which contains chickens, turkeys, quail, guineafowl, and pheasants, is the most economically important avian order, and their genetics, physiology, development, and behavior have been studied extensively. Indeed, the first published avian genome was from Red Junglefowl (*Gallus gallus*), the ancestor of domesticated chickens. Several additional galliform genomes have been sequenced [3–6], but none of these genomes are close relatives to the Red Junglefowl. Thus, little is known about genome evolution for the closest relatives of the junglefowl (*Gallus* species). Of particular interest are genomes of the bamboo partridges (*Bambusicola*), the sister genus of *Gallus* [7], with the Chinese Bamboo Partridge (*Bambusicola thoracicus*) being the best-characterized species. *Gallus* and *Bambusicola* diverged from each other in the Miocene [8], approximately 15 million years ago (MYA), while the *Gallus/Bambusicola* clade diverged from the francolins about 20 MYA [8]. Comparative genomic studies between the *Bambusicola* and *Gallus* genera provide an opportunity to study changes in life history traits at the molecular level.

There are a number of phenotypic and behavioral differences between the junglefowl (*Gallus*) and *Bambusicola*. Junglefowl exhibit striking sexual dichromatism. Males are more colorful than females, with elaborated tail, hackle, and saddle feathers. In addition, combs and wattles in males are much larger, and typically redder, than in females (with *Gallus varius* females lacking combs and wattles completely). Males are also larger than females, and frequently possess large spurs used in combat with other males. Although some *Gallus* may nest monogamously, they are frequently polygynous [9]. Sexual selection is well documented in *Gallus gallus,* with females exhibiting strong preferences for males with larger, redder combs [10, 11]. In contrast, the much smaller *Bambusicola* are monochromatic and exhibit little size dimorphism [9]. Although male *Bambusicola* have spurs, which are weapons used in male-male competition in galliforms, they are much smaller (even relative to body size) than Red Junglefowl spurs [12]. Bamboo partridges appear to be monogamous, as they are frequently seen in pairs that often duet [13]. These differences between junglefowl and bamboo partridges imply that different characteristics have been selected for in each lineage.

*Bambusicola thoracicus* and *Gallus gallus* also differ demographically. Although *Gallus gallus* did not have the nearly worldwide distribution of domestic chickens, the historical range of *Gallus gallus* was still relatively large, covering much of southeast Asia, where they were typically found in various habitats going up to 2000 m in elevation [13]. Overall, the estimated historical range size for *Gallus gallus* is 5,100,000 km^2^ [13]. *Bambusicola thoracicus* is the most widespread of the three species of *Bambusicola*, but it still had a much smaller historic range than *Gallus gallus*. Its native range is restricted to China where it has an estimated range size of 1,280,000 km^2^ [14]. Although the historic range sizes suggest that the ancestral population size of *Gallus gallus* was larger than that of *Bambusicola thoracicus*, *Bambusicola* are much smaller than *Gallus*, and small body sizes are associated with larger effective population sizes [15]. Thus, it is not clear which species had a larger ancestral effective population size (*N*_*e*_).

Here we present a draft genome of *Bambusicola thoracicus* as a resource for comparative evolutionary analyses within galliforms, especially with respect to *Gallus*. We explored the impact of demographic and phenotypic change on genome evolution in these two species. Demographically, we considered two mutually exclusive alternative hypotheses (Table 1). First, the larger range size of the Red Junglefowl reflects a larger *N*_*e*_ in that species, and therefore, we should observe a lower nonsynonymous to synonymous substitution rate ratio (*dN/dS*) in *Gallus gallus* compared to *Bambusicola thoracicus.* Alternatively, the smaller body size of *Bambusicola thoracicus* may have led to a larger historic *N*_*e*_, leading to a smaller *dN/*dS compared to *Gallus gallus*. The expectation of a lower *dN/*dS in whichever taxon had the larger *N*_*e*_ reflects the greater efficacy of purifying selection in large populations. However, strong natural and sexual selection also could have affected patterns of genome evolution, leading to two additional hypotheses that are not mutually exclusive (Table 1). Bamboo partridges appear more similar to francolins, the sister taxa of the common ancestor of *Bambusicola* and *Gallus*. Francolins are largely monogamous with little dichromatism, and they do not exhibit elaborate ornamental traits. If the phenotypic differences between *Gallus* and *Bambusicola* reflect strong directional selection (either natural or sexual), we predict an elevated *dN/dS* due to positive selection in *Gallus*, at least for a proportion of genes associated with phenotypes under selection. Additionally, if sexual selection has been more intense in the *Gallus gallus* lineage, we expect *Gallus gallus* to exhibit lower heterozygosity across the genome than *Bambusicola thoracicus*.

**Table 1:** Predicted hypotheses for rates of molecular evolution in *Bambusicola* and *Gallus*

	Hypothesis	Mean *dN/dS*	*dN/dS* > 1	Heterozygosity
Demography (assuming no selection)	Higher *N*_*e*_ in *Gallus*	Lower in Gallus	Higher in *Gallus*	Higher in *Gallus*
	Lower *N*_*e*_ in *Gallus*	Higher in Gallus	Lower in *Gallus*	Lower in *Gallus*
Selection (assuming equal *Ne*)	Directional selection on *Gallus*	Higher in *Gallus*	Elevated for a proportion of genes in *Gallus*	Equal between species
	Sexual Selection on *Gallus*	Higher in *Gallus*	Elevated for a proportion of genes in *Gallus*	Lower in *Gallus* (due to reduced male population size)

Predicted patterns of molecular evolution and heterozygosity for *Gallus* compared to *Bambusicola* are given for both demographic and selection-based hypotheses of genome evolution. The mean *dN/dS* refers to results for gene-wide differences in molecular evolution from branch tests while *dN/dS* > 1 is determined by results from branch-site tests

We assembled a draft *Bambusicola thorasicus* genome sequence using a single Illumina library with 25× coverage. In addition to testing the proposed biological hypotheses, we addressed technical questions regarding the use of relatively low-coverage genomes generated using short-read technologies. Specifically, we assessed multiple approaches for assembling the *Bambusicola* genome from such data and evaluated the quality of our assembly using both a standard BUSCO analysis [16] and an assessment of our ability to recover an independently generated set of curated ultraconserved elements (UCEs) from our genome assembly [17]. Given the interest in identifying selection in many comparative genomic studies, we determined whether alignment errors could have an impact on estimates of *dN/dS*. Our evaluation of the de novo *Bambusicola thoracicus* genome demonstrates that relatively low-coverage bird genomes can provide valuable evolutionary insights and that they allow the annotation of genes as well as large-scale genomic comparisons.

## Methods

### Genome sequencing, assembly, and annotation

The *Bambisicola thoracicus* sample came from blood preserved in lysis buffer obtained from a female individual from a captive breeding program, and the sample was originally collected for Kimball et al. [18]. This individual was released back to captivity after blood collection. We extracted DNA using the Gentra PureGene DNA Isolation Kit (Qiagen) following manufacturer’s instructions. Mitochondrial regions previously sequenced from this individual [18] showed high identity to published sequences sampled throughout the range [19], indicating it was correctly identified. Library preparation, fragment selection, and sequencing was carried out at the University of Florida Interdisciplinary Center for Biotechnology Research. A single insert library was prepared using Illumina’s NEBNext Ultra DNA Library Prep Kit following the manufacturer’s instructions. A single insert of approximately 500 bp was selected for the NextSeq500 sample preparation protocol. The library was sequenced on a single flow cell using paired-end 150 bp reads on an Illumina NextSeq500.

We assembled a *Bambusicola thoracicus* draft genome sequence using de novo methods. First, we discarded PCR duplicates from the genome sequencing reads using in-house Perl scripts. Then we removed Illumina barcodes and adapters using Trimmomatic [20]. We only retained sequences with an average Phred score of at least 20, with each four-base sliding window having an average Phred score of at least 15, and with a minimum sequence length of 70. We corrected possible sequencing errors based on the distribution of Kmer frequencies using SOAPec v2.01 with default settings [21]. We then built de novo assemblies from the edited reads using SOAPdenovo2 v2.04 [21] and ABySS v1.9.0 [22]. We also assembled the genome with MaSuRCA v2.3.2 [23], which uses its own raw data quality control tools. For computational feasibility, the three assemblies used Kmer values of 63, 63, and 35 respectively, and we merged scaffolds with Metassembler v1.5 [24]. We estimated proportions of repetitive elements with RepeatMasker v4.0.5 using the “Aves” repeat library [25].

We annotated scaffolds with a length of 1 kb or greater using MAKER v2.31.8 [26] and conducted gene predictions using AUGUSTUS v3.2.1 [27] with a hidden Markov model trained from *Gallus gallus* RefSeq sequences. In addition to ab initio gene prediction, we used homologous protein evidence for annotated genes from the amino acid sequences of *Gallus gallus* [28], *Meleagris gallopavo* [29], and *Taeniopygia guttata* [30]. We assessed annotation quality with BUSCO v1.1b1 [16], which estimated the proportion of genes missing from our annotations.

To account for possible annotation biases in downstream analyses, in which we compared our *Bambusicola* data with other galliform genomes, we reannotated the genomes of *Coturnix japonica* [4, 31] and *Colinus virginianus* [5, 32] using MAKER [26] as described above. Since the draft genomes of *Coturnix japonica* and *Colinus virginianus* also are based solely on computational predictions, this step makes the gene models for the three draft genomes in this study more comparable than if they were generated using different prediction methods. We did not reannotate *Gallus* or *Meleagris* because the assembly and annotation for both of those genomes is excellent. In fact, a number of gene models have been validated using RNAseq data.

Finally, we assessed both the assembly quality and our ability to accurately annotate unique sequence features in the *Bambusicola thoracicus* genome using 3854 ultraconserved elements (UCEs) that were generated by sequence capture from the same individual [17]. The UCE sequences were aligned against the masked *Bambusicola* assembly using the NUCmer program [33] from MUMmer [34] with default settings. We extracted coverage and mismatch information for each UCE from the pairwise alignments.

### Pairwise comparisons between Bambusicola and Gallus

We assessed the completeness of our assembly by comparing it with the reference *Gallus gallus* genome assembly [35]. We aligned the masked *Bambusicola* scaffolds to *Gallus* genomic sequence using the NUCmer [33] with default settings. We calculated the proportion of sequenced bases, pairwise nucleotide diversity, and GC content for non-overlapping 100 kb sliding windows from the alignable sequences using Perl scripts. Results from sliding windows were visualized with CIRCOS [36].

### Testing hypotheses of molecular evolution

We circumscribed gene families from the annotated genomes of *Gallus gallus*, *Bambusicola thoracicus*, *Coturnix japonica*, *Meleagris gallopavo*, and *Colinus virginianus* using OrthoMCL [37], and we identified those gene clusters that had exactly one sequence from each of the five species. We refer to these as *the single-copy orthologous groups*. We obtained codon alignments for the single-copy orthologous groups by first aligning amino acids with MUSCLE [38] and then mapping codons onto the multiple sequence alignments with a custom Perl script (https://github.com/gtiley/Alignment_Tools/tree/master/Codon_Alignment). We constructed maximum likelihood gene trees for all single-copy orthologous groups with all five taxa present from the codon alignments using RAxML [39] with the GTR + Γ nucleotide substitution model. We attempted to improve the accuracy of the gene tree topologies using TreeFix [40]. TreeFix uses the species tree, the ML estimate of the gene tree, and the multiple sequence alignment as input, and it searches for the rooted gene tree topology that implies the fewest duplications and losses without a significant decrease in the likelihood compared to the ML tree. For the TreeFix analysis, we used the species relationships from Hosner et al. [41]; however, the relationships among our focal taxa are strongly corroborated by multiple data types [7, 42].

We next attempted to identify and remove anomalous or erroneous gene sequences that might mislead subsequent molecular evolution analyses from the single-copy orthologous groups. We optimized *dN*/*dS* on each branch of the gene trees using PAML v. 4.8a [43] and calculated the minimum patristic distance based on *dS* for each sequence in the single-copy gene trees using the ape package [44] in R [45]. We constructed a Beta distribution from the mean and variance of the minimum patristic *dS* estimates. We removed any sequence that had a nearest neighbor distance in the 99th percentile of the theoretical Beta distribution from the gene tree and excluded the gene tree from further analyses.

We tested rates of molecular evolution on the remaining single-copy orthologous groups in which the gene tree topology after the TreeFix analysis was identical to the species tree topology (i.e. gene trees in which the sequences appear to be orthologs). We used PAML v. 4.8a [43] to test whether any of these single-copy orthologous groups exhibited shifts in gene-wide *dN*/*dS* [46] and to test for episodic positive selection acting on a proportion of sites [47]. For both tests, we designated a single branch of interest as the foreground branch; the remaining branches are the background branches. In the tests for shifts in gene-wide *dN/dS* (branch tests), the null hypothesis was that all branches of the gene tree had the same *dN/dS* whereas the alternative hypothesis added one additional free *dN/dS* parameter on the foreground branch. For branch tests, the *p*-value can be calculated using the likelihood ratio test (LRT), assuming that the LRT statistic is approximately distributed as χ^2^_1_. The test for episodic positive selection (branch-site test) similarly used a LRT ~ χ^2^_1_, but rather than a single *dN/dS* across all sites in the gene, it modeled molecular evolution with finite mixtures of *dN/dS* values across sites. For the null hypothesis, all branches in a gene tree had a proportion of sites under purifying selection (i.e. *dN/dS* < 1) and a proportion of neutrally evolving sites (i.e. *dN/dS* = 1). The alternative hypothesis allowed a third class where a proportion of sites under positive selection (i.e. *dN/dS* > 1) are present on the foreground branch.

We tested three branches in the single-copy orthologous groups for differences in the gene-wide *dN/dS* and for positive selection: 1) the *Bambusicola thoracicus* terminal branch, 2) the *Gallus gallus* terminal branch, and 3) the branch leading to the most recent common ancestor (MRCA) of *Bambusicola* and *Gallus*. These three branches were treated as unconstrained foreground branches for both branch and branch-site tests. Since our analyses involved hypothesis tests for many gene trees, we controlled for false positives by assuming a false discovery rate of 0.05 and applying the method of Benjamini and Hochberg [48]. Briefly, *p*-values were ranked from lowest to highest, and the Benjamini-Hochberg correction computed a *q*-value for each *p*-value that depended only on the false discovery rate and the number of tests performed. If a *p*-value was less than its corresponding *q*-value, we rejected the null hypothesis. This allowed us to reduce the number of false positives while not applying a multiple-testing correction as severe as the Bonferroni [48]. Although branch-site tests can be vulnerable to false discovery rates higher than 0.05, we accepted this correction as a necessary trade-off due to the low power for tests of positive selection using gene trees with only five taxa [49]. We preformed corrections for the *Bambusicola*, *Gallus*, and MRCA branch and branch-site tests independently.

### Uncertainty in estimates of episodic positive selection

While the branch-site test has high power even when only a small proportion of sites have experienced positive selection [50], it may be susceptible to alignment errors [51]. To account for potential alignment error in the branch-site tests, we reanalyzed the single-copy orthologous groups with significant branch-site tests by integrating the branch-site tests over alignment uncertainty using BAli-Phy [52, 53], a program that implements a Bayesian Markov chain Monte Carlo (MCMC) analysis that jointly estimates posterior distributions of the alignment and *dN*/*dS* parameters. For each test, we ran two independent chains of 25,000 MCMC samples and proposed five alignments for each sample, discarding the first 2500 iterations from each chain as burn-in. We applied the Rao-Blackwell estimator for the posterior probability of positive selection. We diagnosed convergence when all parameter posterior distributions had an effective sample size greater than 300 and a potential scale reduction factor of approximately 1 for the 80% credible interval. Complete details of the BAli-Phy analyses are in the supplementary material (Additional file 1).

### Effects of alignment error on estimates of gene-wide *dN/dS*

We also examined whether alignment error affected branch tests. For single-copy gene families that had a significant branch test from PAML, we ran BAli-Phy for one chain of 10,000 iterations under a codon model with a single *dN/dS* across all branches and randomly sampled 100 alignments without burn-in, as the MCMC chain started with the MUSCLE alignment (Additional file 1). We then re-evaluated the branch tests on these 100 alignments using PAML with methods described above.

### Enrichment of functional categories in the genes subject to selection

We assigned GO terms to each single-copy orthologous group based on *Gallus gallus* annotations from AgBase [54]. Enrichment for generic GO slim processes, functions, and components [55] were inferred from genes with significant branch or branch-site tests using a two-sided Fisher exact test implemented in R [45]. We tested for overrepresentation and underrepresentation of GO terms based on the number of significant and non-significant tests for a branch of interest compared to all other significant and non-significant tests. For example, the contingency table when testing for enrichment of a GO category on the *Bambusicola* branch would have ((*Number of significant tests for GO category in Bambusicola*, *Number of significant tests for GO category not in Bambusicola*), (*Number of nonsignificant tests for GO category in Bambusicola*, *Number of nonsignificant tests for GO category not in Bambusicola*)) (Additional file 1: Figure S1). For branch tests, we treated orthologous groups that had a *dN/dS* significantly higher or lower than the background separately. Again, we controlled for a false discovery rate of 0.05 with a Benjamini-Hochberg correction [48].

### Estimating long-term effective population size and measuring heterozygosity

In order to explore how demographic differences between *Bambusicola thoracicus* and *Gallus gallus* may explain the observed patterns of molecular evolution, we estimated effective population size (*N*_*e*_) over time for *Bambusicola* using the Pairwise Sequential Markov Coalescent (PSMC) model [56]. We used SAMtools mpileup on a BWA [57] alignment of *Bambusicola thoracicus* paired-end reads to their de novo genome assembly to call heterozygous bases, but applied the C50 option to correct mapping quality scores for high quality reads with uncertain mapping positions, such as repetitive regions of the genome. All genomic sites considered for PSMC analyses had a minimum read depth of 10 and a maximum read depth of 50. Because of the reduced recombination on sex chromosomes, we only analyzed the genomic sequence data that mapped to autosomes. Since genome architecture of birds is relatively stable (e.g. [58, 59]), we excluded those *Bambusicola* scaffolds that mapped to the Z chromosome from the NUCmer alignment with the *Gallus* reference genome. Scaffolds that did not uniquely align to any *Gallus* autosome also were excluded from PSMC analysis.

To compare the *N*_*e*_ estimates over time from the *Bambusicola* genome with those from *Gallus gallus*, we reanalyzed two *Gallus gallus* genomes from Wang et al. [60] that had 23× and 35× coverage. We downloaded data from GenBank BioProject accession PRJNA241474 [61] for SRA accessions SRX511214 and SRX511217 respectively. Short reads were trimmed and corrected using methods described above. We generated two PSMC analyses for the *23× Gallus* genome: 1) Short reads were aligned to the reference *Gallus* genome using BWA [57], and 2) a de novo assembly was generated using MaSuRCA [23] and reads were then aligned to the de novo assembly with BWA [57]. We had insufficient computer memory to construct a de novo assembly for the 35× genome. We called heterozygous bases using the same methods as for *Bambusicola*, except the 35× *Gallus* genome had a minimum read depth of 15 and a maximum read depth of 70. Again, we used only data that mapped to *Gallus gallus* autosomes.

We ran all PSMC analyses using 64 atomic time intervals with the options –N 30 –t 15 –r 5 –p “4 + 25*2 + 4 + 6”, such that the recombination rate was relatively constant across each atomic time interval. The PSMC model of Li and Durbin [56] compressed heterozygosity information into bins by examining 100 bp of contiguous sequence and calling a bin heterozygous if there was at least one high quality bi-allelic site in within those 100 bp. We further split the binned sequence data into segments of no more than 10,000 bins and performed 100 bootstrap replicates for each analysis to measure uncertainty in estimates of *N*_*e*_. We assumed a generation time of 1 year [62, 63] and a mutation rate of 1.91 × 10^− 9^ per year [64] for both *Bambusicola* and *Gallus* to convert from coalescent units to absolute time.

In addition to inferring differences in *N*_*e*_ based on the distribution of heterozygous sites, we wanted to explore differences in heterozygosity across autosomes between *Bambusicola* and *Gallus*. We estimated the proportion of heterozygous sites per chromosome using the mpileup calls on our *Bambusicola* scaffolds that could be aligned to the reference *Gallus gallus* genome. We only considered bi-allelic sites with a depth of at least 20, bases with phred scores of 20, and a minor allele frequency of at least 0.1. We estimated a false positive rate for our heterozygous base calls using 21 independently Sanger sequenced nuclear introns from the same *Bambusicola thoracicus* individual [42, 65]. For comparison, we aligned masked scaffolds from the 23× *Gallus* de novo assembly to the reference *Gallus* genome and called heterozygous bases as previously described.

## Results

### Bambusicola assembly and annotation

The paired-end NextSeq run of a single 500 bp insert library resulted in 116 million reads, with approximately 100 million reads retained from the forward and reverse read after quality filtering (Additional file 2: Table S1). The merged assembly resulted in 163,749 scaffolds with an N50 of 13,160 bp. The total length of the assembly was 1.03 Gb, with a minimum scaffold length of 218 bp, a maximum scaffold length of 311 kb, and a median scaffold length of 3145 bp (Table 2). Assembly statistics varied considerably among de novo assembly methods, and the merged assembly most closely resembled the MaSuRCA results (Table 2). The assembly consisted of approximately 11% repetitive content, with the largest component, 6.71%, consisting of CR1 LINES (Table 3). The overall level of repetitive content in *Bambusicola* is similar to *Gallus*, and greater than the other galliform genomes we examined (Table 3).

**Table 2:** Comparison of *Bambusicola* assemblies and published genomes utilized in this study

	SOAP denovo2	ABySS	MsSuRCA	*Bambusicola*	*Colinus*	*Coturnix*	*Gallus*	*Meleagris*
Total contig length	1.475 Gb	1.049 Gb	1.068 Gb	–	–	–	–	–
Total scaffold length	1.543 Gb	1.049 Gb	1.069 Gb	1.032 Gb	1.172 Gb	1.751 Gb	1.047 Gb	1.062 Gb
# contigs	3,657,399	872,000	255,563	–	–	–	–	–
# scaffolds	2,514,856	871,673	232,161	163,749	220,307	275,637	15,932	5884
# Contigs > 1 kb	188,688	194,673	145,400	–	–	–	–	–
# Scaffolds > 1 kb	312,353	194,682	128,638	123,582	65,748	275,637	14,828	5866
Med. contig length	254	162	1383	–	–	–	–	–
Med. scaf. Length	282	162	1296	3145	559	2991	1206	1561
Max contig length	97.41 kb	111.0 kb	201.7 kb	–	–	–	–	–
Max scaf. Length	400.4 kb	111.0 kb	311.0 kb	311.0 kb	600.7 kb	1.501 Mb	195.3 Mb	204.1 Mb
Contig N50	508	5668	10,589	–	–	–	–	–
Scaffold N50	1154	5670	12,505	13,160	45,461	11,408	90.22 Mb	74.86 Mb
Gene Models	–	–	–	17,772	15,984	27,554	16,354	16,494
Comp. BUSCOS	–	–	–	45%	54%	46%	90%	81%
Frag. BUSCOS	–	–	–	19%	18%	20%	4%	7%

Assembly statistics for individual methods are given as well as the merged *Bambusicola* assembly. Comparative assembly metrics for other genomes included in this study were taken from primary literature

**Table 3:** Relative abundances of interspersed repeats across genomes

	*Bambusicola*	*Colinus*	*Coturnix*	*Gallus*	*Meleagris*
Total	9.51%	5.69%	5.17%	9.64%	7.67%
Retroelements	8.44%	4.92%	4.37%	8.58%	6.74%
SINEs	0.08%	0.06%	0.07%	0.08%	0.07%
CR1 LINEs	6.71%	4.06%	3.76%	6.79%	5.67%
ERV LTRs	1.64%	0.80%	0.53%	1.71%	1.00%
DNA transposons	1.02%	0.73%	0.75%	1.01%	0.88%
hobo/Activator	0.53%	0.38%	0.36%	0.53%	0.45%
Tc1/Mariner	0.29%	0.21%	0.21%	0.30%	0.26%
Tourist/Harbinger	0.04%	0.03%	0.03%	0.03%	0.03%
Unclassified	0.05%	0.04%	0.05%	0.05%	0.05%

Percentages are notable repetitive element content for genomes included in this study. All estimates were generated with RepeatMasker v4.0.5

**Fig. 1:**
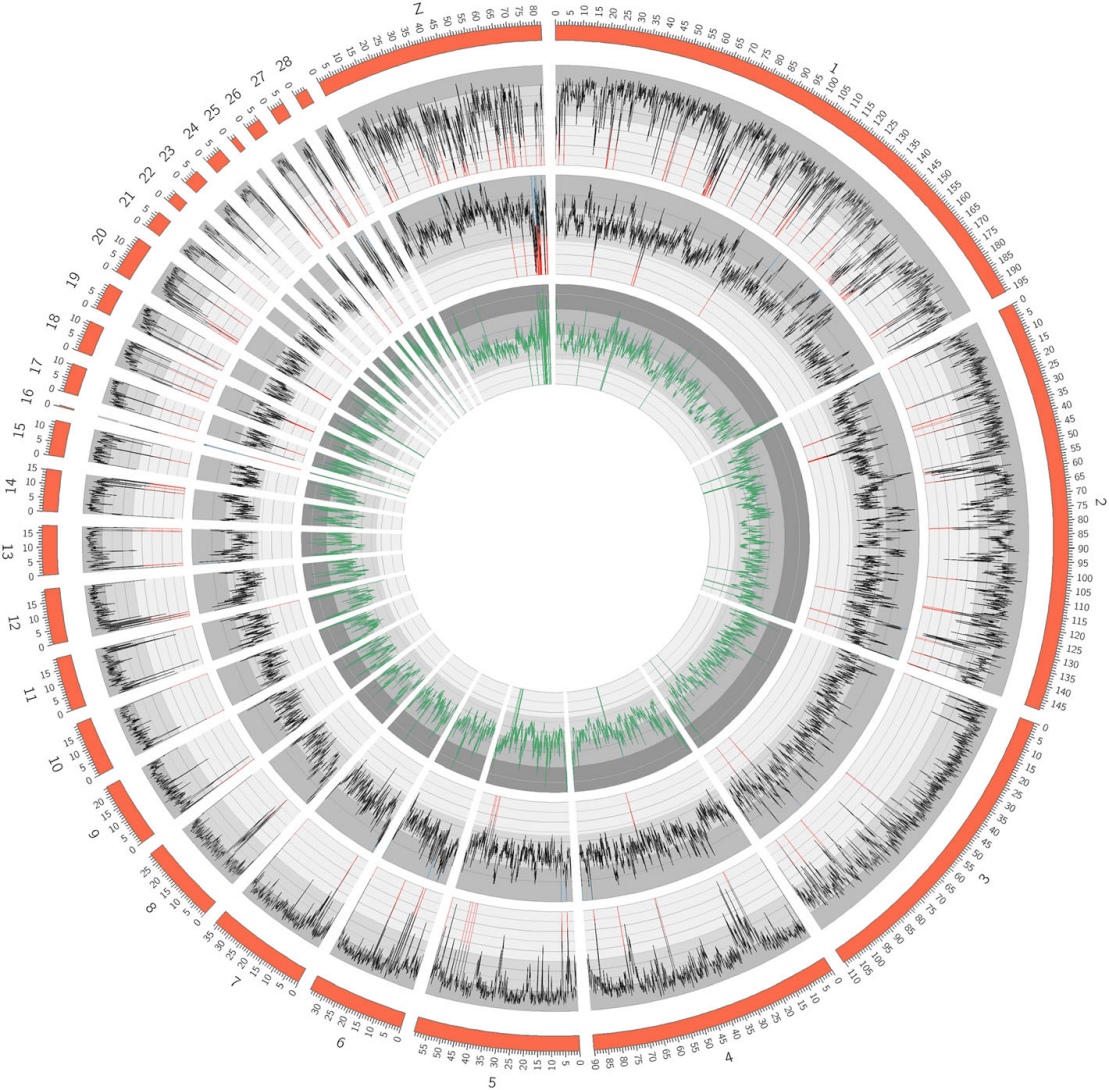
Circular plot of the *Gallus gallus* genome sequence assembled at the chromosome level compared with our *Bambusicola thoracicus* genome. Analyses were across non-overlapping 100 kb sliding windows. The outermost line plot represents breadth of coverage of *Bambusicola* with respect to *Gallus*. Bases aligned by NUCmer were considered sequenced while all gaps were considered missing data. Only values between 50% and 100% are shown. All areas where the breadth of coverage drops below 50% are drawn in red. The middle line plot shows pairwise nucleotide divergence. Missing data and gaps were excluded from this estimate. Only values between 0.02 and 0.08 are shown. Values below 0.02 are shown in red while values above 0.08 are shown in blue. The innermost ring is variation in GC content. The purple line is *Gallus* GC content and the green line is *Bambusicola* GC content. Only values between 20% and 60% are shown. Chromosomes 8 through 28 are magnified in Additional File 1 (Figure S3)

We assembled approximately 90% of the *Bambusicola* genome based on scaffolds aligned to the reference *Gallus gallus* genome (Additional file 2: Table S2). While the whole-genome alignment to the *Gallus gallus* genome suggests we assembled 85%–95% of most chromosomes, our scaffolds could not be aligned to 47% of chromosome 16, 32% of chromosome 25, and 29% of the Z chromosome (Additional file 2: Table S2). Pairwise comparison between the *Bambusicola* and *Gallus* NUCmer alignment also revealed local assembly gaps in the *Bambusicola* genome (Fig. 1). We expected these portions of the genome to be poorly assembled, especially chromosome 16, which contains the rapidly evolving MHC loci, many members of the large olfactory receptor gene family, and repetitive regions such as LTRs, LINEs, and ribosomal DNA repeats (e.g. [66]).

Among the assembled portion of the *Bambusicola* genome, we achieved an average of 25× coverage, given the distribution of kmer frequencies as well as the depth of short read alignment to the *Bambusicola* de novo assembly (Additional file 2: Tables S1 and S2). We observed an average pairwise nucleotide divergence of 5.4% between *Bambusicola* and *Gallus*; however, pairwise nucleotide divergence varied across the genome and appeared to be especially high in some regions with low coverage (Fig. 1; Additional file 1: Figures. S2 and S3). These regions of the *Bambusicola* genome were likely difficult to align to the *Gallus* genome. GC content across the genome appeared relatively consistent in the 100 kb windows between *Bambusicola* and *Gallus*, but the GC content was generally higher on the microchromosomes (Fig. 1; Additional file 1: Figure S3), as has been noted in other studies (e.g. [67]).

MAKER annotations produced 17,772 gene models in the *Bambusicola* genome, with 44% coverage of core vertebrate orthologs from BUSCO. Our new annotations of the *Coturnix japonica* and *Colinus virginianus* genomes found 27,544 and 15,948 gene models, with 46% and 54% coverage of core vertebrate orthologs respectively. For comparison, *Gallus gallus* and *Meleagris gallopavo* cover 90% and 80% of vertebrate single-copy orthologs from BUSCO respectively (Table 3).

Out of the 3854 reference UCE sequences from the same *Bambusicola* individual [17], 3842 were present in the masked assembly. The average assembly coverage of a UCE was 98.7%, and the average frequency of mismatches per UCE was 0.09%. The mismatches could represent sequencing errors, assembly errors, or missed heterozygous base calls. 2390 of the UCEs were represented in the *Bambusicola* assembly at full length with no mismatches between our genome assembly and the previously sequenced UCEs. If we allowed mismatches between the *Bambusicola thoracicus* UCE sequences and our genome assembly, there were 3172 full-length UCEs represented in the *Bambusicola thoracicus* genome. Overall, most previously sequenced UCEs from the same *Bambusicola thoracicus* individual were represented in our assembly with high similarity (Additional file 1: Figure S4).

When we clustered the amino acid sequences from all five genomes, we identified 15,864 orthologous groups, 3285 of which had a single-copy from all five species after removing potentially erroneous sequences. Among these single-copy, putatively orthologous groups, 2822 had gene tree topologies that were identical to the species topology following the TreeFix analysis. From these 2822 gene trees, we identified a subset of 374 orthologous groups with high sequencing accuracy, such that all sites in the *Bambusicola* sequence had at least 20× depth with all phred scores at least 14. This subset of 374 orthologous groups were analyzed with the total set of 2822 gene trees and separately to observe if sequencing depth and base qualities might influence overall patterns of molecular evolution in our study (Additional file 1).

### Variation in dN/dS – Branch tests

**Fig. 2:**
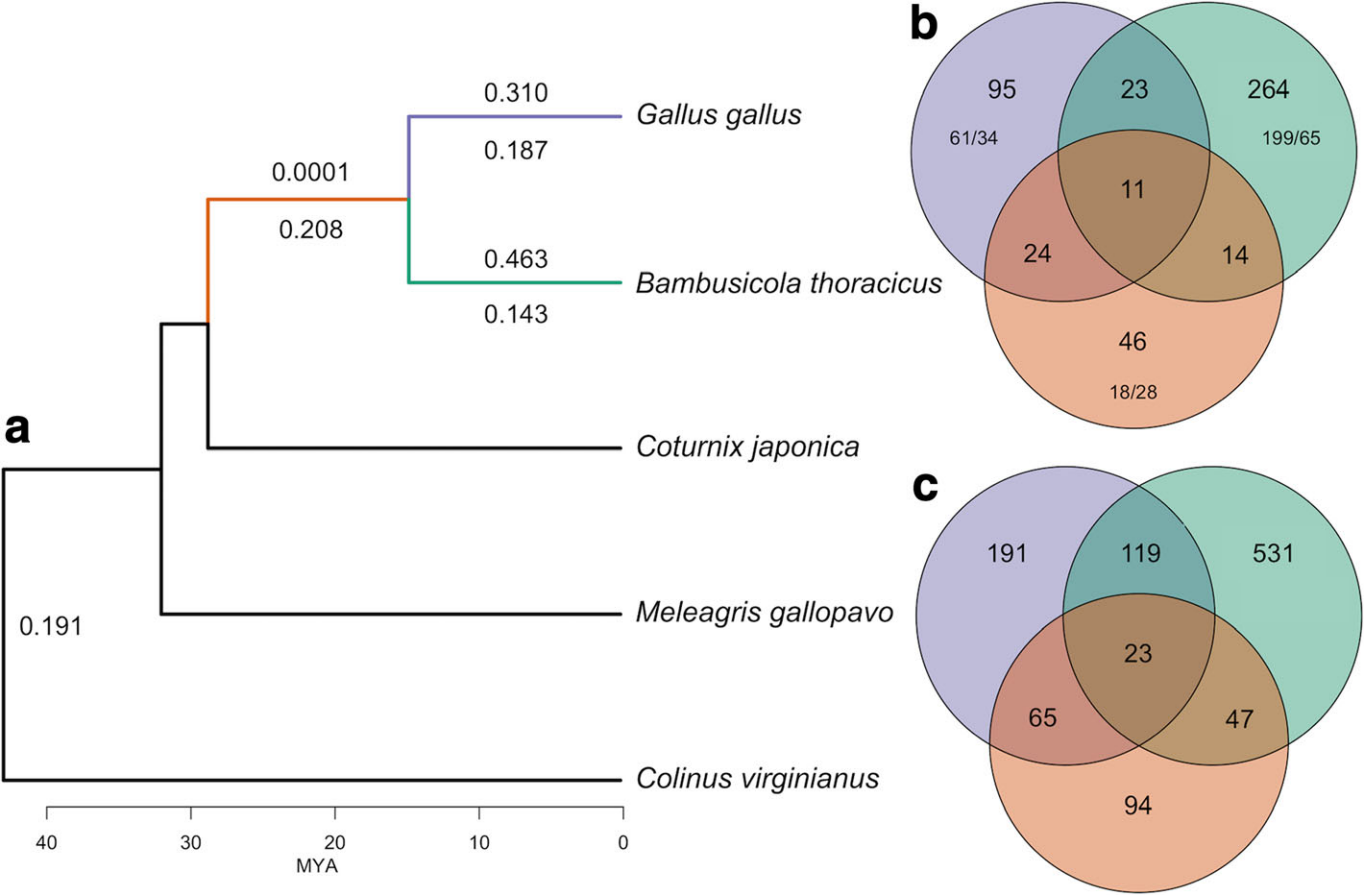
Significant branch and branch-site tests on branches leading to *Gallus*, *Bambusicola*, and their most recent common ancestor. Colors correspond to the foreground branches in the branch and branch site tests. **a**) Numbers above foreground branches are the median foreground *dN/dS* for significant branch tests; numbers below branches are median background *dN/dS* for significant branch tests (i.e. the *dN/dS* on the other branches in the tree). **b**) The number of significant branch tests on the three foreground branches. Branch tests that were significant in only one foreground branch were classified into genes that are evolving with a higher *dN/dS* than the background rate / genes that were evolving at a lower *dN/dS* than the background rate. **c**) The number of significant branch-site tests across foreground branches, suggesting potential positive selection

**Fig. 3:**
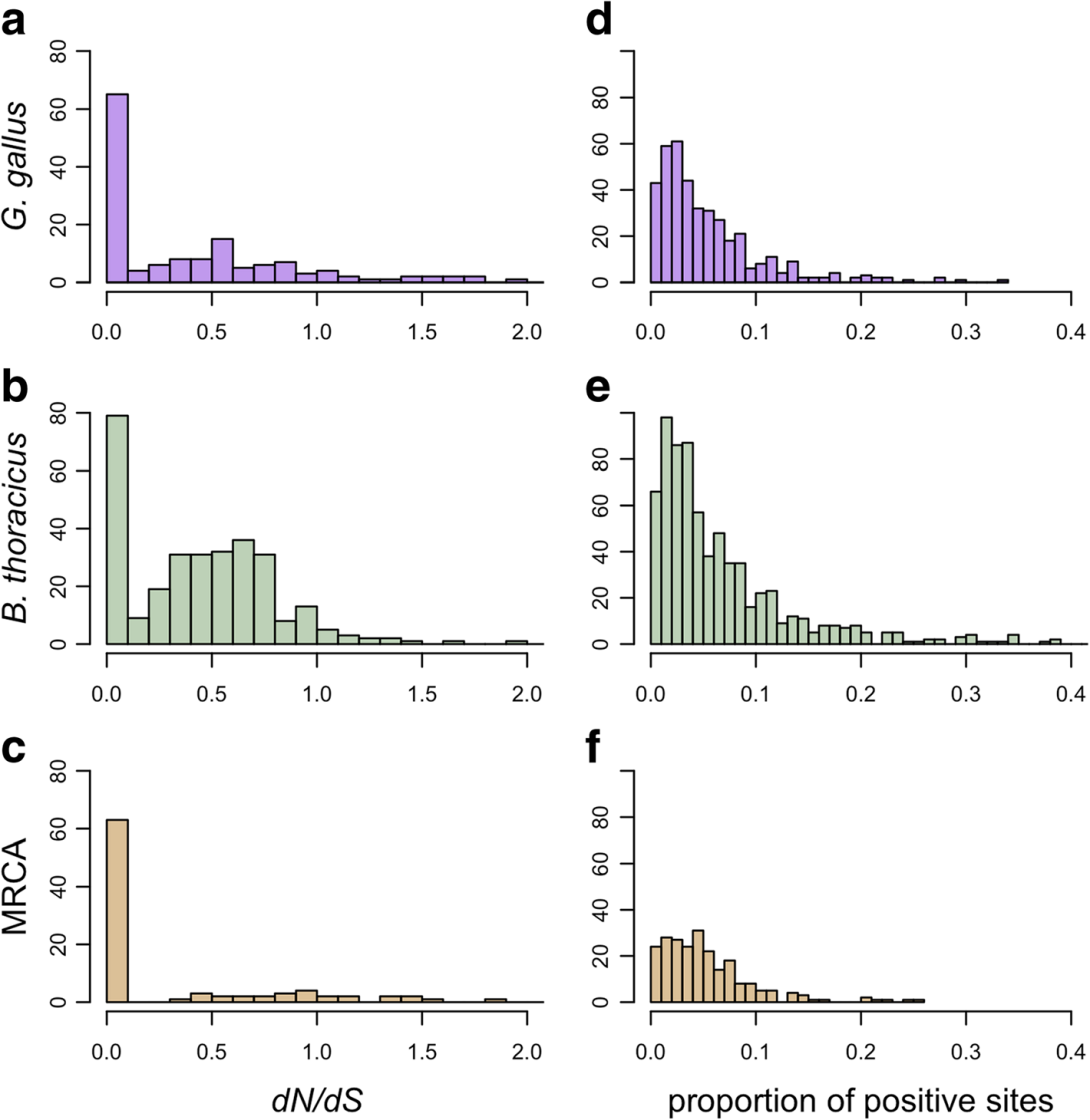
Distributions of foreground gene-wide *dN/dS* and the ML estimates of the proportions of sites under positive selection for significant branch and branch-site tests. Results are after multiple-testing corrections. *dN/dS* distributions were truncated at two for ease of visualization and because excessively high *dN/dS* estimates likely are unreliable. Panels **a**-**c** correspond to gene-wide *dN/dS* from branch tests while panels **d**-**f** correspond to the proportion of sites under positive selection from branch-site tests

Branch tests revealed many genes suggesting relaxed selective pressures (i.e. significantly higher *dN/dS* than background branches) in both the *Bambusicola* and *Gallus* lineages, and strong purifying selection (i.e. significantly lower *dN/*dS than background branches) in the branch leading to their MRCA (Fig. 2a; Table 4). There were more significant branch tests, indicating a different *dN/dS*, in *Bambusicola* (11.1% of the genes) than in *Gallus* (5.4% of the genes). 75% and 64% of significant branch tests indicated an increased *dN/dS* in *Bambusicola* and *Gallus* respectively (Fig. 2b; Table 4; Additional file 2: Table S3). Only 1.6% of gene trees indicated a different *dN/dS* on the MRCA branch, with 61% evolving at a lower *dN/dS* compared to the *Bambusicola* and *Gallus* branches. The median foreground *dN/dS* for significant branch tests in *Bambusicola* (0.463) was higher than that in *Gallus* (0.310; Fig. 3a-3c). We also found a similar pattern of more gene trees with elevated *dN/dS* in *Bambusicola* in our subset of 374 orthologous groups (Additional file 1).

**Table 4:** Summary of branch and branch-site tests of molecular evolution

	*Bambusicola*	# trees	*Gallus*	# trees	MRCA	# trees
Branch	264(199)	2822	95(61)	2822	46(18)	2822
resampled *q*	13(9)	264	9(5)	95	2(2)	46
resampled *p*	46(22)	264	24(10)	95	16(6)	46
Branch-Site	531	2822	191	2822	94	2822
BAli-Phy	3	531	2	191	1	94

Results from ML estimates are shown in bold while subsequent methods are shown in plain text below. Resampled *q* refers to the use of *q*-values while *resampled p* refers to the use of *p*-values for distributions of alignments for branch tests. The number of trees tested for each species is given to the right of each respective column. Numbers in parentheses for branch tests are the number of gene trees where the foreground *dN/dS* was greater than the background *dN/dS*. Numbers for branch-site tests represent the number of genes that showed evidence of episodic positive selection

We re-evaluated the branch tests that were significant after multiple-testing corrections across the distribution of alignments created by BAli-Phy [53]. Comparing LRT statistics to each test’s original *q* value, only 0.5%, 0.3%, and 0.001% of the original number of gene trees were still significant on the *Bambusicola*, *Gallus*, and MRCA branches respectively. If we relaxed the multiple testing requirement and considered genes with a *p* value < 0.05 for at least 95% of alignments, we found more significant tests, but there was more evidence of relaxed selection on the *Bambusicola* branch than the other branches in all analyses (Table 4).

### Variation in *dN/dS* – Branch-site tests

We found 31.6%, 14.1%, and 8.1% of the genes in *Bambusicola*, *Gallus*, and their MRCA respectively had some proportion of sites with *dN/dS* > 1 based on branch-site tests (Additional file 2: Table S4). If we only consider genes that were significant in only one of these three branches, the proportion of positive tests was 18.8%, 6.8%, and 3.3% for the *Bambusicola*, *Gallus*, and MRCA branches, respectively (Fig. 2c; Table 4). The distribution of the proportion of sites under positive selection was similar across the *Bambusicola*, *Gallus*, and MRCA branches (Fig. 3d-3f). Our subsampled data also revealed elevated numbers of genes in *Bambusicola* that appear to have experienced episodic positive selection (which we define as those orthologous groups for which including a proportion of sites with *dN/dS* > 1 significantly improves model fit) compared to *Gallus* or the MRCA of *Bambusicola* and *Gallus* (Additional file 1).

**Fig. 4:**
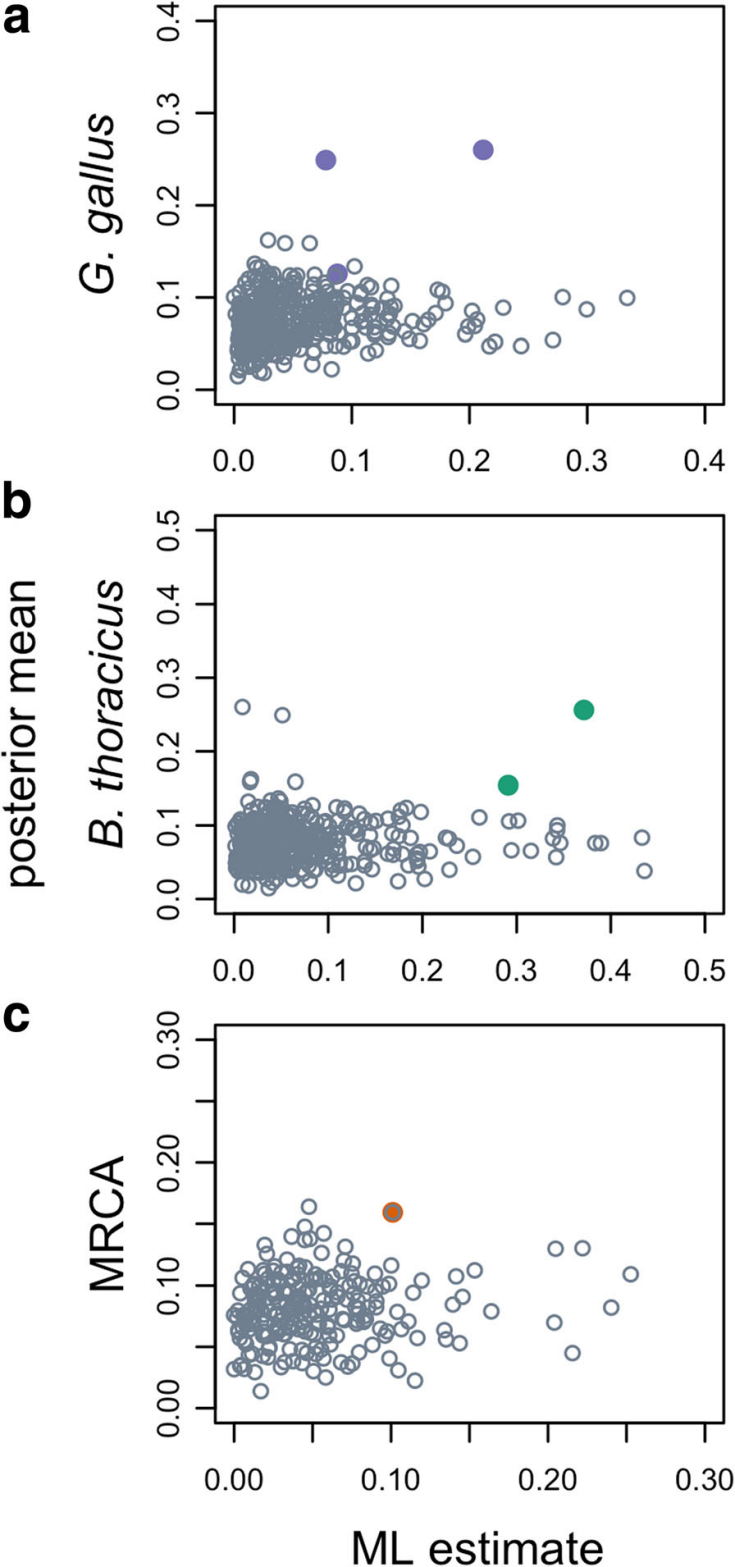
The estimated proportion of sites under positive selection for significant branch-site tests. Significant results are indicated by the LRT after multiple testing corrections. The original ML estimate is from PAML while the mean of the posterior distribution is from post-burnin BAli-Phy estimates. Genes with a posterior probability of positive selection > 0.95 are shown in color while genes with a posterior probability ≤0.95 are shown as empty grey circles. Panels **a**-**c** show the respective distributions from the *Gallus gallus*, *Bambusicola thoracicus*, and their MRCA foreground branches

Alignment error likely contributed to many significant branch-site tests. Posterior probabilities of positive selection from BAli-Phy only supported 3 genes with sites under positive selection on the *Gallus* branch, 2 on the *Bambusicola* branch, and 1 gene on the MRCA branch (Fig. 4; Table 4). There was a weak association between the proportion of sites under positive selection from both the ML and Bayesian estimators for the *Gallus* branch (Kendall’s τ = 0.125; *p* < 0.001) and the *Bambusicola* branch (Kendall’s τ = 0.201, p < 0.001), but not the MRCA branch (Kendall’s τ = 0.07 *p* = 0.112). Thus, while there is some agreement between the two estimators on the proportion of sites with *dN/dS* > 1, there is much uncertainty in the inference of episodic positive selection (Fig. 4). In most of our analyses using MUSCLE alignments for which MLEs imply positive selection, there also are possible alignments that do not imply positive selection.

For the MUSCLE alignment results, there was some overlap between the significant branch and branch-site tests. For the *Bambusicola* branch, 224 genes were significant for both tests, with 19 of these having a gene-wide *dN/dS* > 1 (Additional file 2: Tables S3 and S4). On the *Gallus* branch, 81 genes had significant branch and branch site tests (Additional file 2: Tables S3 and S4), including 23 genes with a global *dN/dS* > 1. The MRCA branch had an additional 28 genes with significant branch and branch site tests, with 10 genes having a global *dN/dS* > 1.

### Enrichment of GO categories

**Fig. 5:**
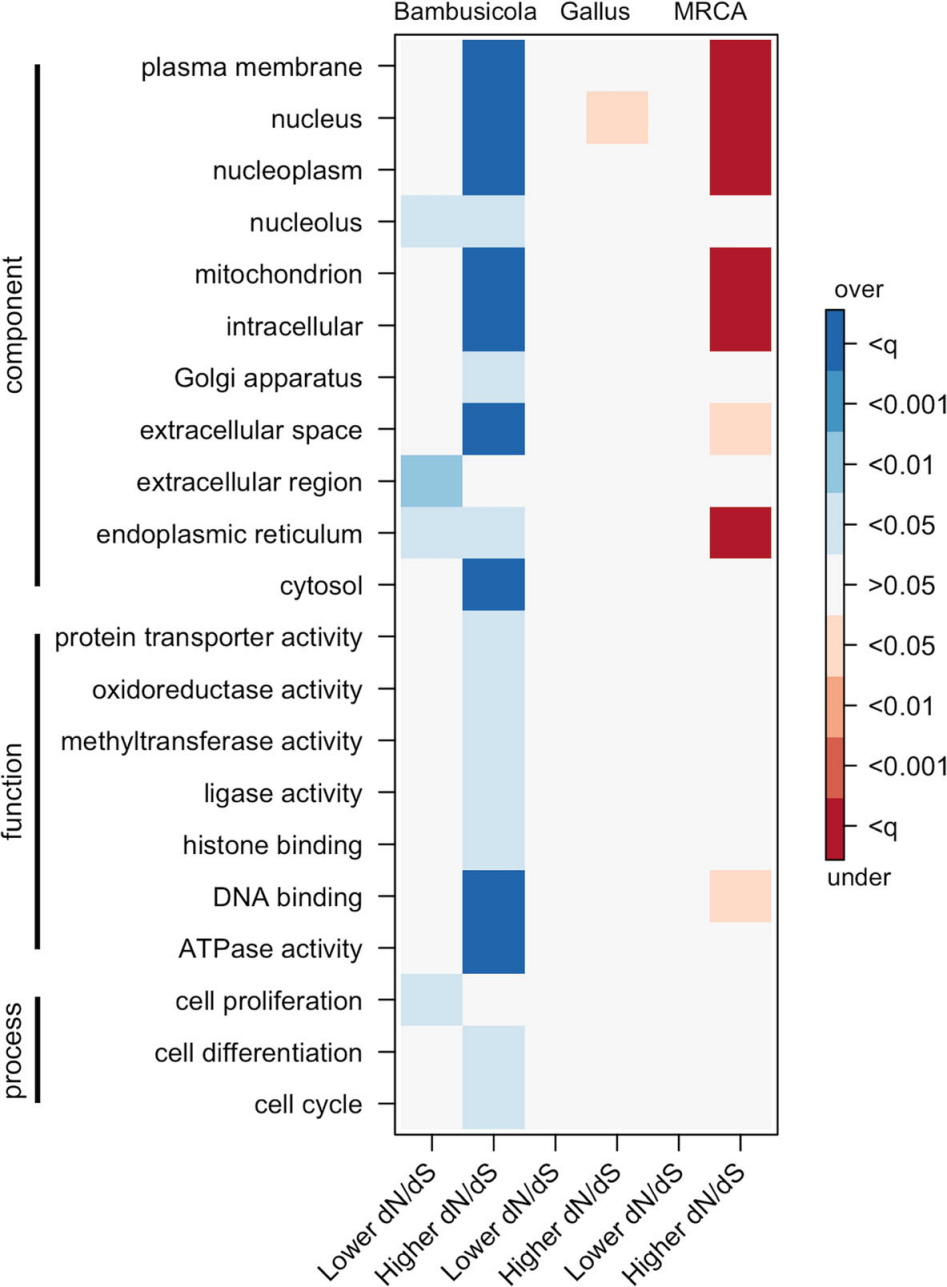
Overrepresentation or underrepresentation of GO slim categories. Tests were conducted for a proportion of sites under positive selection, lower gene-wide *dN/dS*, and higher gene-wide *dN/dS*, on the *Bambusicola*, *Gallus*, and MRCA branches. Overrepresented terms are blue, and underrepresented terms are red. Levels of significance are shown for *p* < 0.05, *p* < 0.01, *p* < 0.001, and *p* < *q*, where *q* is the Benjamini-Hochberg *q*-value corrected for a FDR of 0.05

There were seven cellular components and two molecular functions significantly overrepresented with higher *dN/dS* in the *Bambusicola* branch, while 6 components were underrepresented for increased *dN/dS* in the MRCA branch (Fig. 5; Additional file 2: Table S5). For five components, there was both an overrepresentation of increased *dN/dS* in *Bambusicola* and a simultaneous underrepresentation of increased *dN/dS* in the MRCA. Overall, we tested 127 GO categories, and none were over or underrepresented in *Gallus*. Many GO categories were not relevant due to small numbers represented in our sample of orthologous genes, but the results supported an increase in *dN/dS* in *Bambusicola* across many different GO categories that was not observed in the MRCA or *Gallus* branches.

### Estimating heterozygosity and *N*_*e*_

**Fig. 6:**
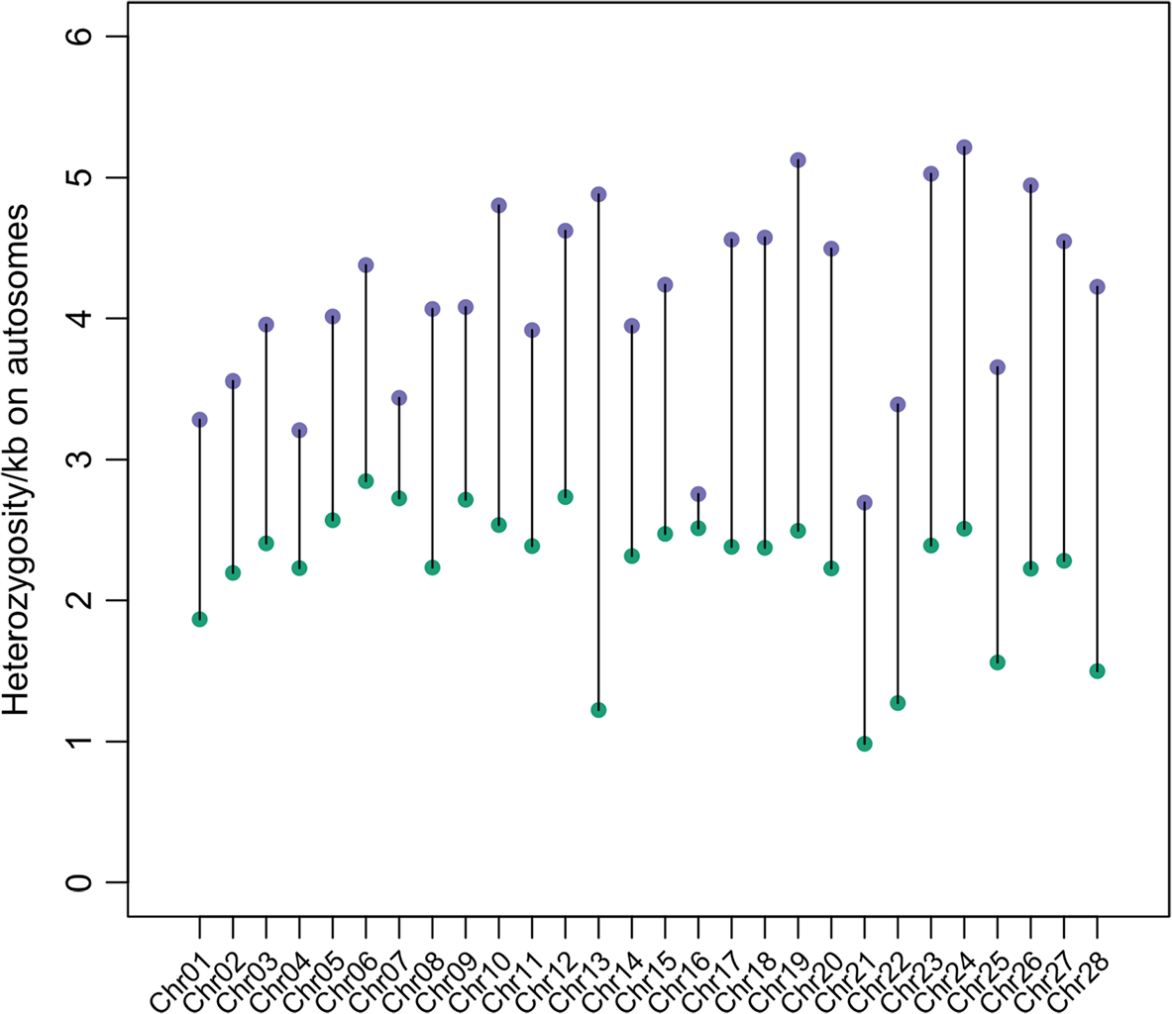
Heterozygosity measured as the number of heterozygous bases per one kilobase. Purple circles represent *Bambusicola* while green circles represent *Gallus*. Base calls were performed on assembled contigs that were alignable to the *Gallus* reference genome, and we only consider the length of these alignable contigs in our heterozygosity estimates. Heterozygosity was higher in *Bambusicola* than *Gallus* for all chromosomes

We identified 2,968,298 heterozygous bases across 781,975,390 total bases of *Bambusicola* genomic data that were alignable to *Gallus* autosomal sequence, representing 3.80 heterozygous bases per 1 kb. The *Gallus gallus* reference alignments of 23× and 35× had 3.80 and 6.36 heterozygous bases per 1 kb respectively; however, there were 1,814,172 heterozygous bases across 806,203,193 bases of autosomal scaffolds in the 23× de novo *Gallus* assembly, representing 2.25 heterozygous bases per 1 kb. Thus, the de novo assemblies estimated lower heterozygosity compared to aligning reads directly to the Galgal4 reference genome, but comparison of heterozygosity for the *Bambusicola* and *Gallus* de novo assemblies alone implied higher heterozygosity in *Bambusicola* (Fig. 6; Additional file 2: Table S6). Evaluation of Sanger sequenced intron data for *Bambusicola* suggested a false positive rate for heterozygous bases of 0.23%, which was consistent with previous benchmarking datasets for Illumina sequencing technology with SAMTOOLS variant calling methods [68, 69].

**Fig. 7:**
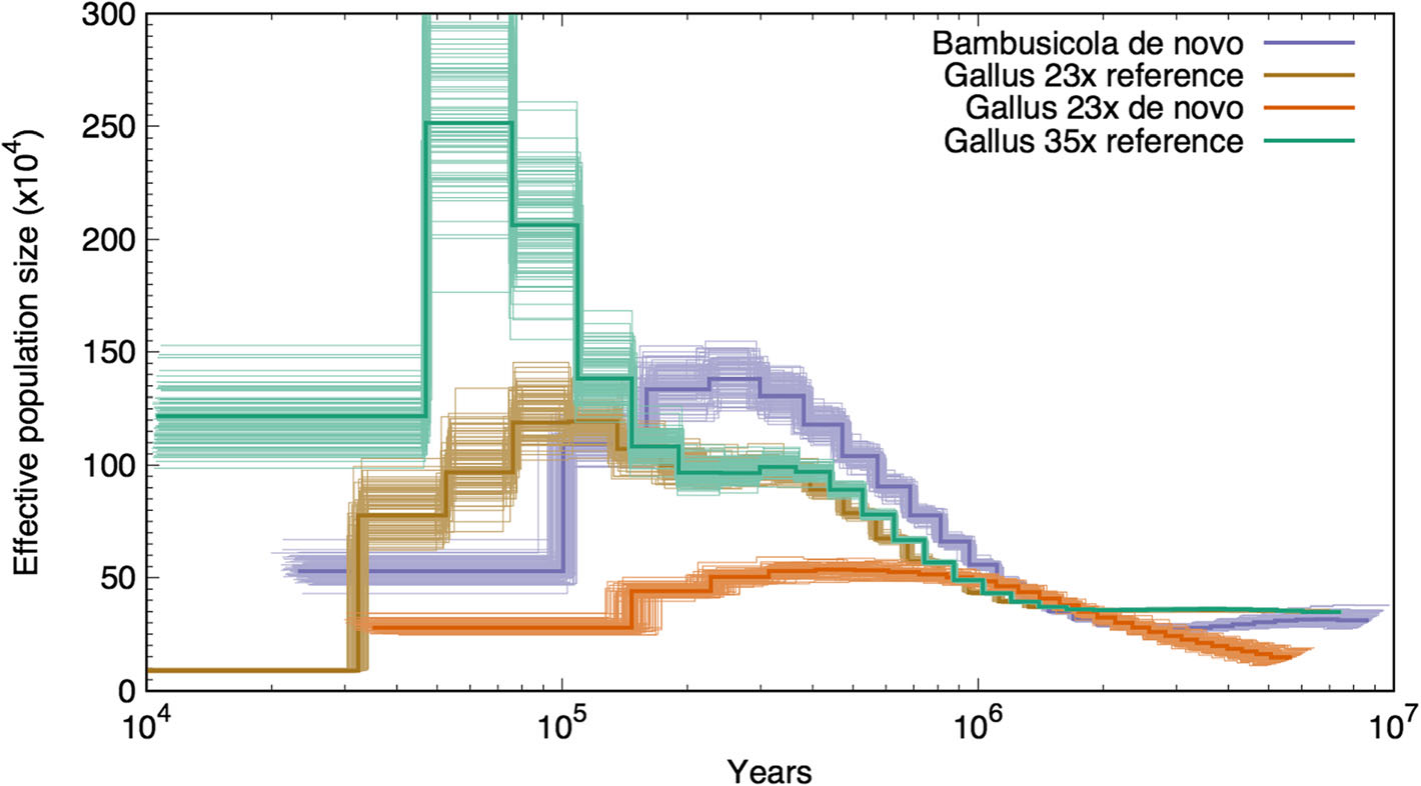
Estimation of *N*_*e*_ over time, inferred from heterozygous bases of individual genome sequences with the PSMC model. Results are shown for the observed data (bold lines) and bootstrap replicates for the *Bambusicola* genome sequence as well as two previously published *Gallus gallus* genome sequences. Estimates are included for the 23× and 35× *Gallus* genomes for short reads aligned directly to the *Gallus* reference genome (reference) as well as short reads aligned to a de novo assembly (de novo) for the 23× *Gallus* genome

PSMC analysis suggested that *Bambusicola* reached its maximum *N*_*e*_ between 100,000 and 500,000 years ago (Fig. 7). Both the *Gallus* 23× and 35× referenced aligned data suggested a maximum *N*_*e*_ in *Gallus* closer to 100,000 years ago (Fig. 7). However, using heterozygosity data inferred from a reference genome or a de novo assembly affected the PSMC results. The *Gallus* 35× referenced data indicated the highest maximum *N*_*e*_. In contrast, the 23× *Gallus* de novo assembly inferred a much older and smaller maximum *N*_*e*_ than that inferred when the data were aligned to the Galgal4 reference assembly (Fig. 7). Although comparisons of the *Gallus* 35× referenced data with the de novo *Bambusicola* genome indicated *Gallus* had a higher maximum *N*_*e*_ than *Bambusicola*, comparison of the de novo *Bambusicola* genome with the de novo 23× *Gallus* genome implied a larger maximum *N*_*e*_ in *Bambusicola*.

## Discussion

Our results demonstrated it is possible to generate an informative draft avian genome from a single library with only moderate coverage for relatively little expense. Complete avian genome sequences are becoming increasingly common [1, 4–6], and the technical issues explored here are not unique to our genome assembly. We attempted to reduce assembly errors by merging multiple de novo assemblies, and pairwise comparisons with the closely related *Gallus gallus* reference genome suggested that we assembled at least 85% of the nuclear genome. Often the most variable regions of the *Gallus* genome, such as chromosome 16, did not align with our *Bambusicola* assembly. However, this is common for bird genomes [60], and it is not unique to low-coverage genome assemblies. Although we had limited success assembling long scaffolds, the size of most *Bambusicola* contigs was comparable to other avian genome sequencing projects using two or more insert libraries with similar (or even higher) coverage [e.g. 1]. We identified and recovered unique sequence features such as protein-coding genes, UCEs, transposable elements, and heterozygous sites with considerable completeness and accuracy. Notably our estimates of repetitive content were consistent with other galliforms, including those sequenced using Sanger sequencing (Table 3). A de novo assembly using short-read next generation sequencing might be expected to underestimate repetitive content, which suggests we have excellent capabilities to assemble contigs of genomic sequence data using single insert libraries.

Our goal was to leverage the *Bambusicola* genome to test molecular evolution hypotheses relevant to the divergent life histories of *Bambusicola* and *Gallus*. Our genome-level analyses of molecular evolution, even when controlling for sources of error, supported elevated *dN/dS* in *Bambusicola* (Fig. 2; Fig. 3). This result was consistent with the hypothesis that the reduced range of *Bambusicola* relative to *Gallus* has led to a lower *N*_*e*_ in *Bambusicola*, and thus relaxed purifying selection over time. We also found increased levels of episodic positive selection in *Bambusicola* with respect to *Gallus*, which was, in contrast to the elevated *dN/dS* results*,* consistent with predictions for a lower *N*_*e*_ in *Gallus.* However, the latter result could also be driven by alignment uncertainty, which likely inflated the error rate for our branch-site tests (Fig. 4). In our case, the use of BAli-Phy greatly reduced the perception of rampant positive selection in the *Bambusicola* and *Gallus* lineages relative to our original branch-site tests that used fixed alignments.

To further test the hypothesis that *Bambusicola* has a lower *N*_*e*_ than *Gallus*, we used PSMC analyses to estimate changes in *N*_*e*_ for both *Bambusicola* and *Gallus*. Similar to PSMC analyses of other avian genomes [70], our results suggested that *N*_*e*_ for *Bambusicola* and *Gallus* peaked around 100,000 years ago, followed by a reduction near the last glacial maximum (Fig. 7). While it may be difficult to obtain reliable estimates of recent *N*_*e*_ from PSMC analyses, our results indicate that *N*_*e*_ was higher in *Gallus* 50,000–100,000 years ago, which may reflect the larger ancestral range of *Gallus* compared to *Bambusicola* and would be consistent with our observation that *Bambusicola* exhibited globally increased *dN/dS* [13, 14]. However, the PSMC model estimates parameters from contiguous sequence data, and the *Bambusicola* genome assembly consisted of many fragmented scaffolds. Differences in assembly quality can lead to disparities in *N*_*e*_ estimates; this was shown in a recent analysis of flycatcher genomes [71], and it was consistent with our comparison of the 23× *Gallus* de novo and reference aligned genomes (Fig. 7). Although these technical issues make it challenging to interpret the results of PSMC analyses, the fact that two very different lines of evidence (the globally increased *dN/dS* and the PSMC results) similarly indicate a smaller *N*_*e*_ for *Bambusicola* increases our confidence in our general conclusions.

Although our results suggested a lower *N*_*e*_ in *Bambusicola* than *Gallus*, heterozygosity was higher in *Bambusicola*, at least when comparing de novo assemblies of similar sequencing depth (Fig. 6; Additional file 2: Table S6). Heterozygosity likely reflects recent events. Thus, the increased heterozygosity in *Bambusicola* may be due to migration events within the last 100,000 years between Pleistocene glacial refugia [72]. Even if *Gallus* populations experienced similar range shifts and migration following Pleistocene glaciations, a higher growth rate and carrying capacity in *Bambusicola* could explain a more rapid increase in heterozygosity [73, 74]; *Bambusicola* populations could increase more quickly than *Gallus* due to both their smaller mass and more males participating in reproduction. It is plausible that if both *Bambusicola* and *Gallus* both experienced reductions in *N*_*e*_, the *Bambusicola* population could recover faster than *Gallus,* which would experience more inbreeding. Thus, there are a number of scenarios that could explain both globally increased *dN/dS* and heterozygosity in *Bambusicola*.

We also attempted to infer if phenotypic differences between *Bambusicola* and *Gallus* were associated with elevated *dN/dS* in specific genes by testing for enrichment of GO terms. Although interpretation of GO enrichment analyses can be difficult given the large numbers of uncharacterized genes and protein products (Additional file 2: Table S7), we found an overrepresentation of a few specific GO terms within the *Bambusicola* lineage, with little to no bias in the *Gallus* lineage (Fig. 5; Additional file 2: Table S5). The overrepresentation of GO terms in *Bambusicola* was largely driven by transcription factors involved in DNA repair and cell cycle pathways, but structural protein-coding genes under the terms such as cytoplasm (GO:0005737), extracellular space (GO:0005615), and plasma membrane (GO:0005886) suggest some additional potentially biologically important changes. For example, we found both a proportion of sites under positive selection and globally elevated *dN/dS* for a serpin peptidase inhibitor ortholog (Entrez Gene: SERPINB5). Given the developmental importance of ovalbumin for birds (e.g. [75]), further investigations of serpin proteins in *Bambusicola* may be warranted. Despite the enrichment of a few specific terms, there is no obvious biological pattern to the enrichment patterns, further supporting that our observations of elevated *dN/dS* in *Bambusicola* are mostly likely due to demographic effects.

## Conclusions

Our analyses highlight that a draft avian genome assembled using a single library can produce high quality data and evolutionary insights. We revealed globally relaxed selective pressures acting throughout the *Bambusicola* genome, which are likely due to demographic effects, such as a lower *N*_*e*_ and smaller range in *Bambusicola* compared to *Gallus*. *Bambusicola* also exhibits high heterozygosity with respect to *Gallus*, which may be due to a combination of post-glaciation migration events and mating system differences. The *Bambusicola* genome can serve as a resource for testing the effects of sexual selection and mating systems on molecular sequence evolution in future studies.

Our *Bambusicola* genome assembly also addressed a number of technical questions. Although the *Bambusicola* genome is fragmented, especially when compared to some recent avian draft genomes based on multiple libraries [1], we recovered similar numbers of gene models and contig N50 statistics as other avian genomes. Our results also highlighted some uncertainty in results from the popular branch-site test due to sensitivity to data and alignment quality. This has broad implications for other similar studies, as the branch-site test is frequently used in genome-level scans for positive selection (e.g. [76–78]). Moreover, the limitations of the branch-site test that we noted are not necessarily specific to low-coverage genome assemblies because they reflect intrinsic features of protein sequence evolution. Single library genome assemblies may sacrifice assembly quality, but the low cost of these assemblies could permit the collection of genome sequences for a larger number of taxa. If the goals of a study do not require large scaffolds or other large-scale structural information, the approach we used could have benefits. Overall, the insights from our *Bambusicola* draft genome outweigh limitations from a fragmented genome assembly, and additional single library genome sequences may prove a valuable and cost effective resource for comparative genomics, molecular evolution, and phylogenetics of birds.

## Additional files

Additional file 1: **Figures S1 – S4.** Details for the sensitivity of *dN*/*dS* analyses to sequence quality and a number of additional analyses are contained within a single pdf. (PDF 5463 kb)

Additional file 2: **Tables S1 – S7.** Additional data from analyses that were too large to fit on a single page are given as Excel speadsheets. (XLS 4168 kb)

Additional file 3: Alignments from Analyses. All alignments and topologies of one-to-one orthologs used for analyses of molecular evolution are given as a single tarball. (TGZ 3336 kb)

## Abbreviations

*dN/dS*: Nonsynonymous to synonymous substitution rate ratio
FDR: False discovery rate
GO: Gene Ontology
LRT: Likelihood ratio test
ML: Maximum likelihood
MRCA: Most recent common ancestor
MYA: Million years ago
*N*_*e*_: Effective population size
PSMC: Pairwise sequential Markov coalescent
UCE: Ultraconserved Element
