# Supplementary material for: Comparison of the Chinese bamboo partridge and red Junglefowl genome sequences highlights the importance of demography in genome evolution
Source: BMC Genomics. 2018 May 8;19:336. doi: 10.1186/s12864-018-4711-0 (PMC5941490; doi:10.1186/s12864-018-4711-0)
Supplement: Supplementary file 1 — Figures S1 – S4. Details for the sensitivity of dN/dS analyses to sequence quality and a number of additional analyses are contained within a single pdf. (PDF 5463 kb) [file 12864_2018_4711_MOESM1_ESM.pdf]

Additional file 1

Comparison of the Chinese Bamboo Partridge and Red Junglefowl genome sequences highlights the importance of demography in genome evolution

GP Tiley[1,2,3], RT Kimball[1,4], EL Braun[1,5], JG Burleigh[1,6]

[1]Department of Biology, University of Florida, Gainesville, FL 32611

[2]Department of Biology, Duke University, Durham, NC 27708

[3]Corresponding author: george.tiley@duke.edu

[4]rkimball@ufl.edu

[5]ebraun68@ufl.edu

[6]gburleigh@ufl.edu

1 Sensitivity of branch and branch-site test to potential sequencing error and sequence quality

Errors in the assemblies and annotations of draft genomes can mislead the molecular evolutionary analyses. Thus, we examined if results from a subset of the *Bambusicola* draft genome with especially high coverage were consistent with the results using the entire draft genome. To identify the subset of genes, we aligned our short read data to the masked *Bambusicola de novo* assembly with BWA [211]. From the SAMtools v1.2 [212] mpileup format, we used custom Perl scripts to identify and extract annotated genes with a depth of at least 20 and a quality score of at least 14 for all bases. We compared the number of significant branch and branch-site tests as well, as the distributions of *dN*/*dS* from branch tests and the proportions of sites under positive selection, from branch-site tests using this high-quality data

subset and the complete data using χ^2 contingency tests and Mann-Whitney U tests with R [208].

There were 264, 95, and 46 significant branch tests for *Bambusicola*, *Gallus*, and the MRCA respectively out of the complete set of 2822 orthologous gene alignments. For the high quality gene set, there were 28, 19, and 17 significant branch tests out of 374 alignments for *Bambusicola*, *Gallus*, and the MRCA respectively. We derived an expected frequency of significant branch tests from the high quality data and calculated the expected number of significant branch tests from the full set of 2822 tests. These frequencies were 28/374=0.075, 19/374=0.051, and 17/374=0.045 for the *Bambusicola*, *Gallus*, and MRCA branches. We observed an excess of significant tests on the *Bambusicola* branch and fewer on the *Gallus* and MRCA branch than expected in the full set of 2822 orthologous groups (χ^2 test = 126.08, $p < 0.001$).

Among the gene families included in the high-quality gene set, 49, 15, and 9 genes possibly underwent positive selection in *Bambusicola*, *Gallus*, and the MRCA respectively, compared to 531, 191, and 94 in the full analyses. Thus, more genes have also experienced possible episodic positive selection in *Bambusicola* than *Gallus*, even when accounting for data quality. However, using the expected frequencies of significant branch-site tests from the high-quality data, there is an excess of positive tests on all three branches tested here in the full set of 2822 orthologous groups (χ^2 test = 136.77, $p < 0.001$).

Thus, sequencing error and assembly, at least in part, may contribute to the number of genes inferred to be experiencing episodic positive selection in addition to variation in gene-wide rates of molecular evolution. However, it is difficult to interpret these results independently of alignment error, which appears to be a much more prevalent problem for testing molecular evolution hypotheses.

2 Enrichment of GO terms across tests of molecular evolution

Tests for enrichment of GO categories have become a ubiquitous practice in comparative genomics and investigations of molecular evolution. We attempted to make coarse connections between the three ontological organizations (Biological Processes, Molecular Function, and Cellular Component) and our significant branch and branch-site tests of molecular evolution. We counted results using Perl scripts and then ran a two-sided Fisher exact test with R as follows:

```
mat <- rbind(c($a,$b),c($c,$d))\n";
x <- fisher.test(mat)\n";
write(paste(x$p.value,"\t",x$estimate),file="temp.out")
```

where:

$a = Number of significant tests in GO term on branch of interest

$b = Number of significant tests in GO term not on branch of interest

$c = Number of non-significant tests in GO term on branch of interest

$d = Number of non-significant tests in GO term not on branch of interest

Figure S1 – Conceptual representation of the contingency table used for Fisher exact tests to investigate over- and under-representation of GO terms. Analyses were conducted for the *Bambusicola*, *Gallus*, and MRCA results independently. GO enrichment analyses were conducted independently for branch tests where *dN/dS* was higher than the background, branch tests where *dN/dS* was lower than the background, and branch-site tests.

	Branch of interest	Branch not of interest
Significant test	$a	$b
Non-significant test	$c	$d

3 Comparative genomics and tests of molecular evolution

Visualization is an important aspect of interpreting comparative genomic results; however, a number of small chromosomes can make scaling difficult. Here we present both the larger and smaller chromosomes in two separate CIRCOS plots to ease visualization. We also show the complete distributions of the synonymous substitution rates (*dS*) for the *Gallus*, *Bambusicola*, and MRCA branches across our 2822 one-to-one orthologous gene trees, which may imply a higher substitution rate, and possibly mutation rate, in *Bambusicola* since the *Gallus* distribution is very similar to the MRCA.

Figure S2 – Circular plot of *Bambusicola* genomic data aligned to the reference *Gallus* macrochromosomes. All chromosomes are shown on a one-to-one scale. Only data mapping to chromosomes 1 through 7 in addition to the Z chromosome are shown.

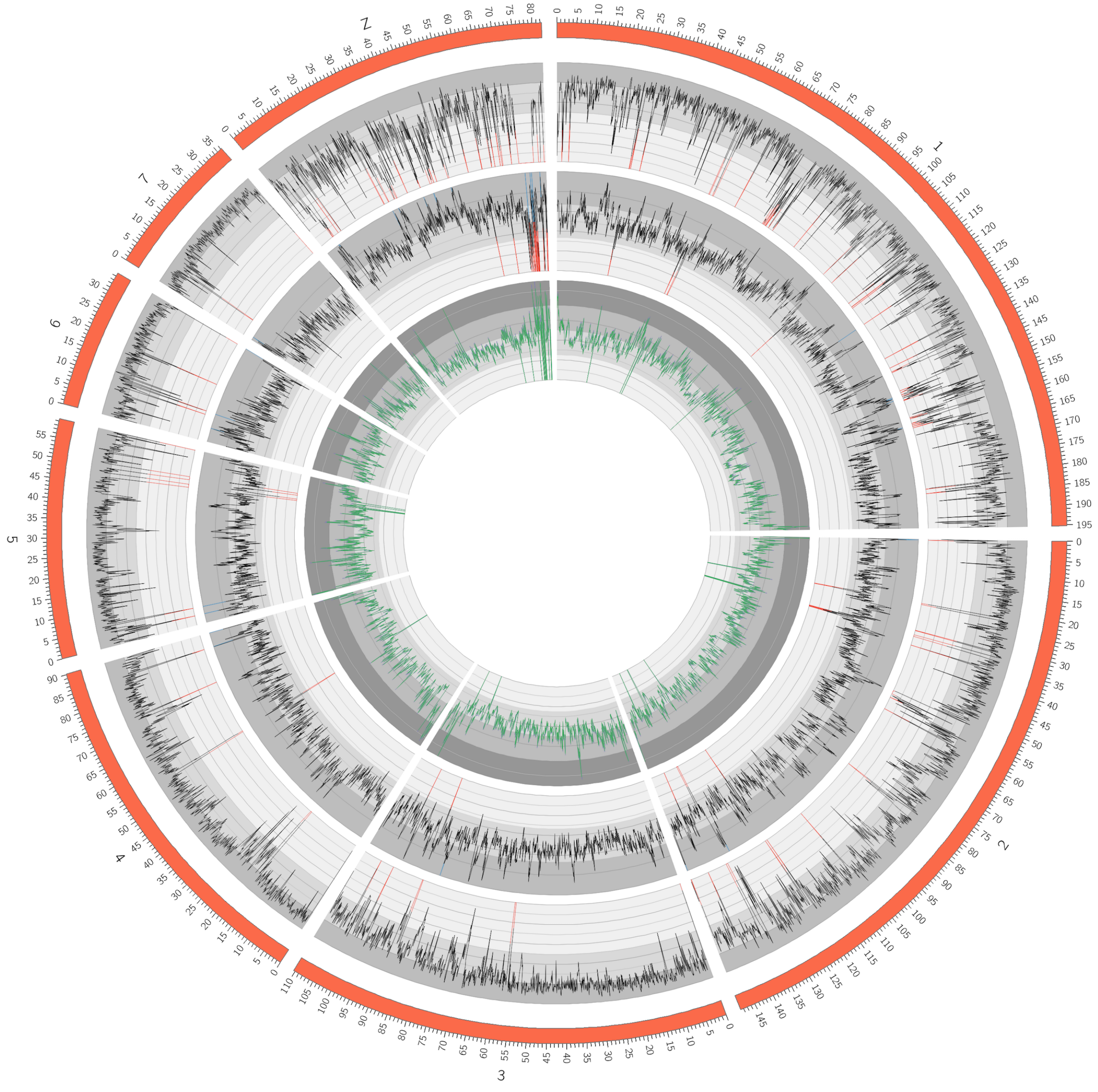

Figure S3 - Circular plot of *Bambusicola* genomic data aligned to the reference *Gallus* intermediate and microchromosomes. All chromosomes are rescaled to occupy 5% of the total plot, to magnify and provide better visualization of smaller chromosomes. Only data mapping to chromosomes 8 through 28 are shown.

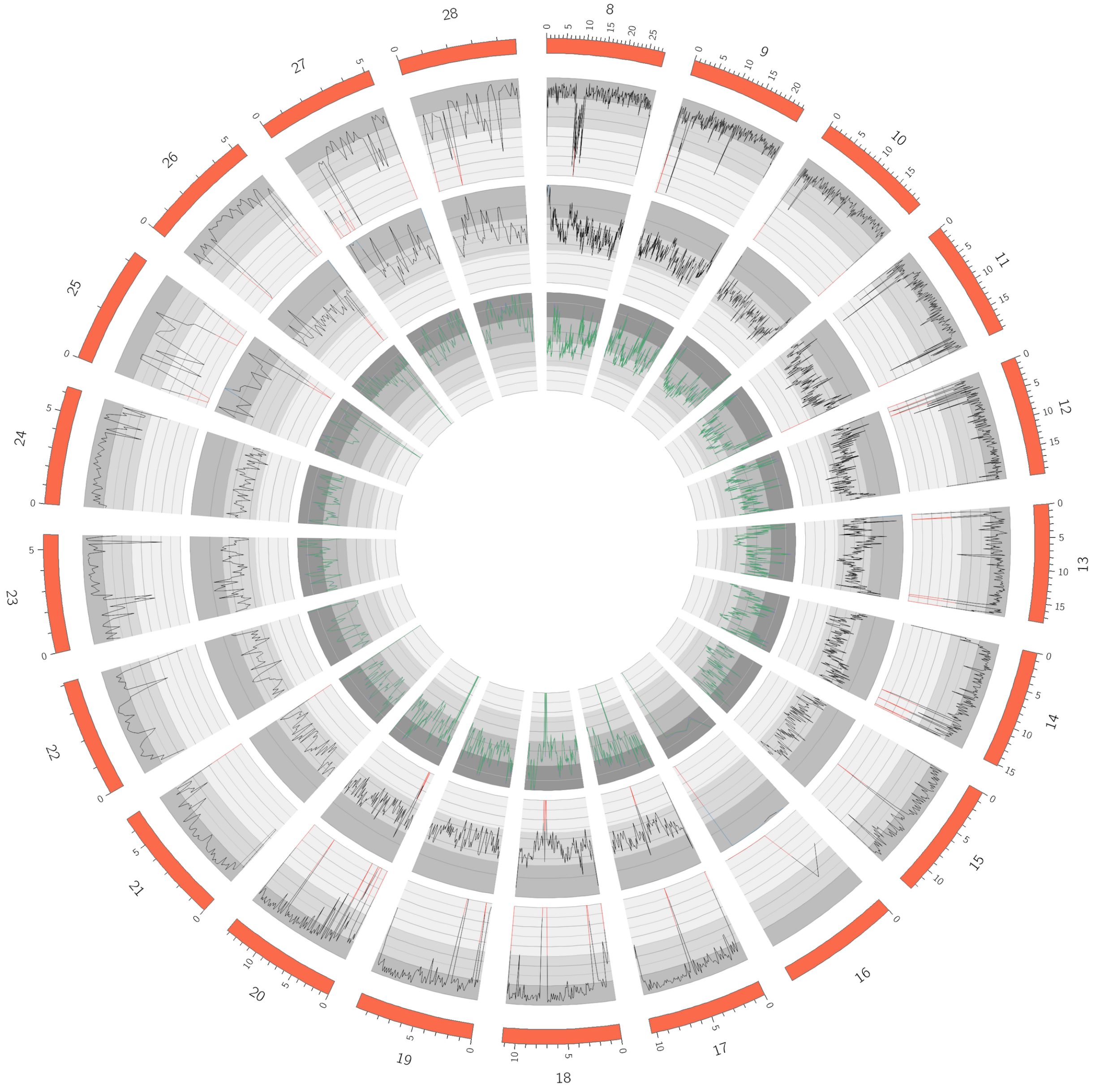

Figure S4 – Distributions of mismatches between previously sequenced UCEs from the same individual with gene capture. Previously sequenced UCEs were aligned to the genomic assembly for *Bambusicola thoracicus*. Most alignments of UCEs with the genome assembly show no mismatches. However, it is possible that only a fraction of the reference UCE sequence was alignable to the *Bambusicola thoracicus* scaffolds (a proportion of assembled UCE < 1) or that the genomic region the UCE aligned to was longer than the reference UCE sequence itself (a proportion of assembled UCE > 1).

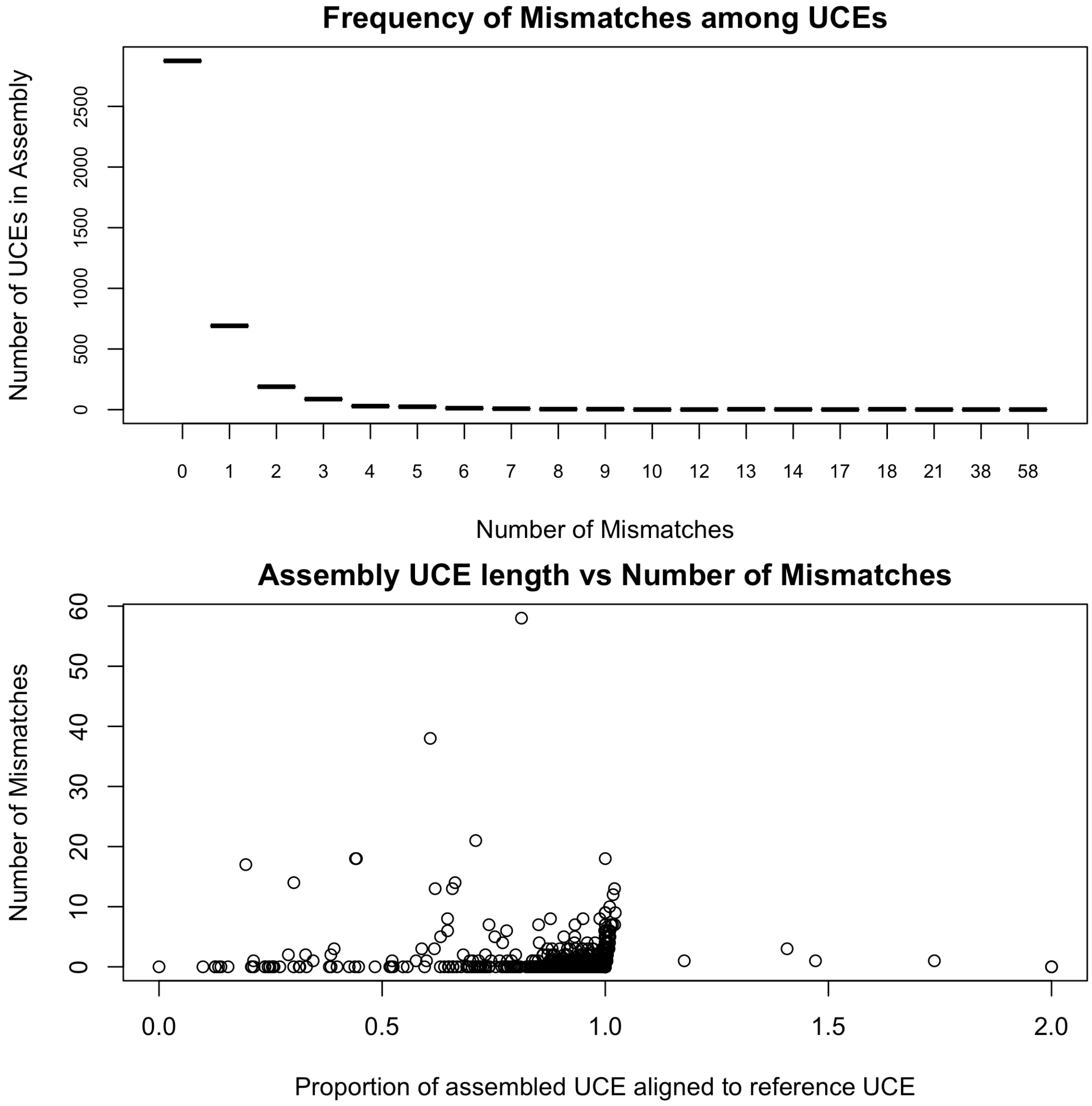

4 Commands to programs used in analyses

4.1 Command to run MUSCLE

```
muscle -in in.aa.fasta -log in.log.txt -out out.aa.muscle.fas
```

4.2 Command to run RAxML

```
raxmlHPC-PTHREADS-SSE3 -f d -s in.codonalign.phy -n
in.codonalign.ml.out -m GTRGAMMA -p int(rand(100000)) -# 10 -T 8 -k
```

4.3 Command to run TreeFix

```
treefix -s speciestree.stree -S speciesIDmap.smap -A .codonalign.fasta
-o .tre -n .treefix.tre -V 0 -l orthogroup.treefix.log orthogroup.tre
```

4.4 Template control file for PAML branch test

Null hypothesis:

```
      seqfile = input.phy
     treefile = input.tre
      outfile = output.txt
        noisy = 0
      verbose = 0
      runmode = 0
      seqtype = 1
    CodonFreq = 2
        model = 0
      NSsites = 0
        icode = 0
    fix_kappa = 0
        kappa = 2
    fix_omega = 0
        omega = 1
```

Alternative hypothesis:

```
     seqfile = input.phy
     treefile = input.tre
      outfile = output.txt
        noisy = 0
      verbose = 0
      runmode = 0
```

```
   seqtype = 1
 CodonFreq = 2
     model = 2
   NSsites = 0
     icode = 0
 fix_kappa = 0
     kappa = 2
 fix_omega = 0
     omega = 1
```

4.5 Template control file for PAML branch-site test

Null hypothesis:

```
   seqfile = input.phy
  treefile = input.tre
   outfile = output.txt
     noisy = 0
   verbose = 0
   runmode = 0
   seqtype = 1
 CodonFreq = 2
     model = 2
   NSsites = 2
     icode = 0
 fix_kappa = 0
     kappa = 2
 fix_omega = 1
     omega = 1
```

Alternative hypothesis:

```
   seqfile = input.phy
  treefile = input.tre
   outfile = output.txt
     noisy = 0
   verbose = 0
   runmode = 0
   seqtype = 1
 CodonFreq = 2
     model = 2
   NSsites = 2
     icode = 0
 fix_kappa = 0
     kappa = 2
 fix_omega = 0
     omega = 1
```

4.6 Command to run BALI-PHY branch-site test (ran twice to get two independent chains)

```
bali-phy input.fasta --alphabet=Codons --smodel=branch-site[,HKY,F3x4]
--disable=topology --tree=input.tre --iter=25000 --Rao-Blackwellize
S1.BranchSiteTest.posSelection --set alignment_sampling_factor=5
```

4.7 Command to summarize two chains from BALI-PHY

```
statreport run1.p run2.p --mean > run.report.mean
```

4.8 Command to run BALI-PHY for sampling alignments for branch tests

In this case, we are not concerned with assessing convergence of model parameters, but only sampling a distribution of alignments. No samples were discarded as burn-in, as the starting alignment was the MUSCLE alignment. Based on the BAli-Phy alignment-sampling algorithm, the following command produces 1000 alignments. We then used a Perl script to randomly sample 100 alignments from the pool of 1000.

```
bali-phy input.fasta --alphabet=Codons --smodel=M0+F3x4[HKY] --
disable=topology --tree=input.tre --iter=10000 --set
alignment_sampling_factor=5
```

4.9 Commands to align reads with BWA and call SNPS with SAMTOOLS

```
bwa index assembly.fasta
bwa mem -t 8 assembly.fasta read1.fq read2.fq > out.sam
samtools faidx assembly.fasta
samtools view -bT assembly.fasta out.sam > out.bam
samtools sort out.bam out.sort
```

```
samtools mpileup -C50 -uf assembly.fasta out.sort.bam | bcftools call
-c | vcfutils.pl vcf2fq -d 10 -D 50 | gzip > allsnps.fq.gz
```

4.10 Commands to align *Bambusicola* scaffolds to *Gallus* with NUCmer

NOTE: Mumer was compiled as **make CPPFLAGS="-O3 -DSIXTYFOURBITS"** to increase the amount of memory available to the program

```
MUMmer3.23/nucmer --prefix=bam galGal4.fa B.A.C.fa.masked
MUMmer3.23/show-coords -rcl bam.delta > bam.coords
MUMmer3.23/show-aligns -r bam.delta > bam.aligns
```
